# Fumigation of Schools for COVID-19 Prevention in Nigeria: The Need for a Rethink

**DOI:** 10.4269/ajtmh.20-1037

**Published:** 2020-08-25

**Authors:** Abisoye Oyeyemi, Adedotun Adesina, Dimie Ogoina

**Affiliations:** 1 Niger Delta University, Wilberforce Island, Nigeria;; 2 Nigerian Law School, Yenagoa, Nigeria

## Abstract

The government of Nigeria ordered closure of schools to slow the spread of COVID-19 when the pandemic hit the country. About 5 months into the outbreak, secondary schools have been reopened to allow students to write their terminal examinations. Many state governments and school owners are fumigating their schools as a way of disinfecting the school environment and ensuring safe resumption of academic activities. We discuss the undue attention given to fumigation in this instance and stress the importance of addressing more beneficial and sustainable strategies to prevent COVID-19 in Nigerian schools.

COVID-19 has continued its unrelenting onslaught on the human race. However, man is fighting back and learning to live with the disease. As a result, many nations are easing lockdown and other restrictive measures enforced to limit movement in addition to implementing and promoting the observance of precautionary measures to slow the spread of the disease.^1,2^ As part of their prevention strategies or in preparation for reopening, governments and organizations in many countries are taking the initiative or are being pressured to invest scarce resources into fumigation of public places as a means of disinfecting and making such environments “safe” against COVID-19.^3,4^

Nigeria ordered secondary schools to reopen on August 4, 2020 after closure for about 5 months. The reopening was to start with students in classes overdue for their terminal examinations.^5^ The Nigeria CDC, in partnership with the Federal Ministry of Education, developed guidelines for schools to ensure safety of students and staff while carrying out academic activities.^6^ The guidelines stipulate a number of essential activities, based on the key strategies of physical distancing, hand hygiene, environmental cleaning, use of face masks, screening, isolation, and notification for further action. Although some state governments are supporting their schools to implement these strategies, others are paying lip service and not doing enough to prepare their schools for the safe resumption of academic activities. What seems to be an obsession of state governments and private school owners across the country in all geopolitical zones is fumigation.^7–11^ Although the national guidelines recommend fumigation as one of the measures to be taken for environmental safety and hygiene,^6^ fumigation seems to have become the cynosure, and it is dwarfing every other recommended measure to fight the disease. But of what value is spraying of classrooms and school premises in the prevention and control of COVID-19?

Infection with SARS-CoV-2, the virus that causes COVID-19, occurs primarily by direct contact with infected persons through their respiratory secretions or respiratory droplets, which may be expelled when infected persons cough, sneeze, talk, or sing.^12,13^ Respiratory droplets that are expelled in this process include the virus and can reach the mouth, nose, or eyes of a susceptible person, and result in infection. The second most important route is fomite transmission, which occurs when hands that touch contaminated surfaces and objects come in contact with the mouth, nose, or eyes.^14^ The virus has been shown to remain viable for hours to days on some fomites and surfaces.^15,16^ The likelihood of environmental contamination is high in healthcare settings where COVID-19 cases are managed, but because there is a possibility of surface contamination outside health facilities, the WHO advises using measures used in treatment centers in other places where large numbers of people gather, including religious houses, markets, and schools.^17,18^ Recommended measures for environmental cleaning and disinfection emphasize wiping potentially contaminated surfaces with cloth soaked in disinfectant solution after cleaning. Cleaning is an essential precursor of disinfection, as it removes or reduces the load of infectious agent, dirt, and other organic matter that may attenuate the potency of the disinfectant.^17^

Fumigation involves spraying disinfectant on surfaces to be decontaminated. The WHO strongly advises against fumigation or direct spraying with disinfectants of humans, as deleterious effects far outweigh benefits.^17,19^ Even in healthcare settings, where fumigation or other “no-touch” methods may be used for terminal disinfection, these are recommended only as supplements and not as a replacement for manual cleaning procedures.^20^ SARS-CoV-2 is transmitted in places where people are active. What purpose is served by fumigating empty school premises that have had no human activities for months? Fumigation in this instance may kill or drive away harmful insects and other dangerous organisms, but there is no scientific evidence to show it to be useful for disinfecting against SARS-CoV-2.

In the face of dwindling financial resources due to the severe economic disruption caused by the pandemic, priority needs to be given to the most beneficial and cost-effective measures to fight the disease. Money earmarked for fumigation should be redirected to ensure implementation of evidence-based strategies recommended for schools. Many schools in Nigeria, particularly public schools, are overcrowded,^21^ and makeshift structures are needed to create additional classrooms to enable physical distancing. Government also needs substantial investment to ensure effective hand hygiene in schools. A recent survey found that only 36% of schools in Nigeria have access to basic water services, 16% to basic water and sanitation services, and 7% to combined basic water, sanitation, and hygiene services.^22^ It is imperative to provide handwashing stations with running water, soap, single-use hand towels, and hand sanitizers at strategic points in schools. More cleaners may need to be employed and additional equipment and items purchased to ensure adequate cleaning of surfaces. Quality fabric masks will need to be provided for staff and students while in school. The schools will need support to make special transport arrangements to ensure students and staff can commute to school safely. Educational materials conveying key messages about COVID-19 prevention should be distributed to be liberally posted to reinforce precautions against the disease. For schools that lack sick bays, this is the time to establish them to ensure prompt attention for any sick student or staff member. In fact, the COVID-19 pandemic offers an opportunity to revamp Nigeria’s weak school health programs.

There are many pressing needs to address to prevent COVID-19 transmission in schools in Nigeria. Fumigation is likely to give a false sense of invulnerability and detract from other more important safety measures. Resources devoted to it should be redeployed to proven, sustainable, and cost-effective strategies that will prevent school outbreaks and help establish a health culture that will outlive COVID-19.
